# Bioelectrical phase angle at diagnosis as a prognostic factor for survival in advanced head and neck cancer

**DOI:** 10.1007/s00405-018-5069-2

**Published:** 2018-07-25

**Authors:** Lars Axelsson, Ewa Silander, Ingvar Bosaeus, Eva Hammerlid

**Affiliations:** 1 Department of Otorhinolaryngology, Head and Neck Surgery, Sahlgrenska University Hospital, Gothenburg, Sweden; 2 Department of Otorhinolaryngology, Institute of Clinical Sciences, Sahlgrenska Academy, University of Gothenburg, Gothenburg, Sweden; 3 Department of Clinical Nutrition, Institute of Medicine, Sahlgrenska Academy, University of Gothenburg, Gothenburg, Sweden

**Keywords:** Head and neck cancer, Bioelectrical impedance analysis, Phase angle, Prognostic factors, Survival

## Abstract

**Objective:**

Bioelectrical impedance analysis (BIA) is used to measure the patient’s body composition, fat-free mass, phase angle (PA), and standardized phase angle (SPA), which are affected by malnutrition. Low values of PA and SPA have been found to be negative prognostic factors for survival in different types of cancer and other severe diseases. The aim of the current study was to investigate whether PA and SPA can be used to predict survival in head and neck (HN) cancer.

**Methods:**

One hundred twenty-eight patients with advanced HN cancer treated in Western Sweden 2002–2006 were examined with BIA at diagnosis, and PA and SPA were calculated. Patients’ age, gender, tumor site, TNM stage, and performance status were obtained, and weight, height, and BIA were measured. Survival up to 12 years was ascertained.

**Results:**

The mean PA was 5.85° and the median was 5.91°. Lower PA and SPA values were significantly associated with shorter overall survival in univariate analyses, together with higher age, oral cancer, higher T class, worse performance status, more weight loss before diagnosis, lower: weight, height, BMI, and reactance. Age, performance status, T class, and PA were significant factors for the overall survival in the multivariable analysis. A PA cutoff value at 5.95° provided the best prediction of 5-year survival.

**Conclusions:**

PA and SPA at diagnosis are significant factors for survival in patients with advanced HN cancer. They are promising prognostic tools to use in treatment planning; further studies are needed.

## Introduction

Head and neck (HN) cancer constitutes approximately 4% of all cancer in Western countries and is more common in developing countries [1]. The average relative 5-year survival for HN cancer is approximately 67% in Sweden, but worse for advanced stages III and IV [2]. The treatments include surgery and/or (chemo)radiation. Many patients with advanced HN cancer have dysphagia and develop malnutrition due to the tumor and its treatment [3, 4]. Malnutrition, measured as unintentional weight loss, has been identified as a negative prognostic factor for HN cancer [5]. A low body mass index (BMI) has also been found to be a negative prognostic factor [6]. Other studies have highlighted the importance of the loss of fat-free mass (FFM) as an important prognostic factor because a loss of muscle mass is considered a key feature of cancer [7, 8].

Bioelectrical impedance analysis (BIA) is an easy-to-use, non-invasive method that measures the electrical properties of the patient’s tissues and has been used for many years to assess body composition including fat-free mass [9]. In recent years BIA has also been used to measure the phase angle (PA), which appears to be even more interesting for cancer survival [10]. PA reflects the amount of water present in tissues and the resistive effects produced by tissue interfaces and cell membranes. Lower PA values suggest reduced cell mass and/or decreased cellular integrity, whereas a higher PA suggests large quantities of intact cell membranes [11, 12]. PA is linked to age, gender, and BMI, and to increase the accuracy, reference values have been published correcting for these factors obtaining a standardized phase angle (SPA) [13, 14]. Low PA and SPA have been associated with shorter survival for some cancers and other serious diseases; however, they have not been well studied for HN cancer [15–17].

The aim of the current study was to investigate whether bioelectrical PA and SPA were predictive for survival in advanced HN cancer.

## Patients and methods

### Study design

One hundred thirty-four patients were treated with curatively intent from 2002 to 2006. These patients participated in our previously published study, where their BIA was evaluated at the time of diagnosis [18]. Measurement problems were encountered in six patients (4%) that resulted in an erroneous BIA value and these patients were excluded, leaving 128 subjects in the study population. The patients’ survival status was followed for as long a period as possible; the median follow-up for the surviving patients was 11.6 years (range 9.4–14.1 years), censor date 8 May 2016. The work described has been carried out in accordance with the code of ethics of the World Medical Association [19]. Ethical approval was received from the Regional Ethics Committee in Gothenburg at the time the original study started in 2002.

### Methods

Patient, tumor, and nutritional data were collected at inclusion before the start of treatment. Age, gender, tumor site, TNM classification, and Karnofsky Performance Status (KPS) were recorded. Nutritional data, including weight and height, were measured, and BMI was calculated. BMI was calculated as weight/height^2^ (kg/m^2^). A BMI in the range of 18.5–24.9 was considered normal weight.

BIA was performed on all patients by a registered dietician (ES) using a Bioelectrical Impedance Analyzer (Model BIA-101S Akern: RJL Systems, Detroit, MI, USA). The patients had fasted for at least 2 h before the measurement. BIA was conducted with the patient lying supine on a bed or exam table, with the legs apart and arms not touching the torso. Evaluations were conducted on the patients’ right side using the standard four surface electrode (tetrapolar) technique on the hand and foot [20]. Resistance (*R*) and reactance (*X*_c_) were measured in Ω at 50 kHz, 800 µA. One assessment of *R* and *X*_c_ was made for each patient, but if no value or an erroneous value was obtained, an additional one to two measurement attempts were made before the patient’s measurement was excluded from further analysis.

FFM was calculated by Lukaski’s equation: FFM = 0.734 (height^2^/*R*) + 0.116weight + 0.096*X*_c_ + 0.878sex − 4.03. Sex: women = 0, men = 1 [21, 22]. Fat-free mass percent was calculated from FFM/weight. Fat-free mass index (FFMI) was calculated as FFM/height^2^ (kg/m^2^). Bioelectrical PA was calculated from the reactance and resistance: PA = arctangent(*X*_c_/*R*) × (180/*π*). SPA was obtained from the PA, using the BMI, age, and gender reference values from the Bosy-Westphal study [13]. Patients with SPA values higher than + 3 SD or lower than − 3 SD were considered outliers with values caused by errors in the impedance measures and these patients were excluded from the study.

### Tumor treatment

Patients were treated according to regional HN cancer guidelines after the decision was made at the hospital’s multidisciplinary tumor conference. Patients with advanced (stages III and IV) oral or unknown primary cancer were treated with surgery, which consisted of excision of the primary tumor, neck dissection, and postoperative radiation. For patients with positive margins or extracapsular tumor extension chemotherapy was added together with the postoperative radiation. Patients with pharyngeal cancer were treated with chemoradiation.

During the study period, radiation was administered according to a standard fractionated schedule of 64.6 Gy for 5 weeks. Chemotherapy usually consisted of induction therapy with two cycles of cisplatin and 5-fluorouracil.

### Statistics

The results are presented as the mean, standard deviation, median, and range for continuous variables and as numbers and percentages for categorical variables. For comparisons between two groups: the Mann–Whitney *U* test was used for continuous variables, the Mantel–Haenszel chi-square test for ordered categorical variables, the chi-square test for non-ordered categorical variables, and Fisher’s exact test for dichotomous variables.

Cox proportional hazard regression analysis was used for univariable survival analysis. Hazard ratios were calculated for descriptive purposes. Forward stepwise Cox proportional hazard regression analysis was used for multivariable survival analysis. Only variables that affected survival time at univariate tests (*p* < .1) were included in the multivariate analysis. A Kaplan–Meier plot was used to describe the overall survival for study subgroups, and the difference between subgroups was analyzed with a log-rank test. A receiver operating characteristic (ROC) curve was used to analyze the association of predicted probabilities and observed responses. Area under the curve (AUC) was calculated for each ROC curve.

All significance tests were two-tailed and conducted at the 5% significance level. SAS, System Version 9.4 (SAS Institute, Inc, Cary, NC, USA), was used for all statistical analyses.

## Results

### Characteristics of the patients

The study population consisted of 128 patients with advanced oral, pharyngeal, or unknown primary cervical cancer (Table 1). The mean age for all patients was 61 years and two-thirds were males. Approximately half the patients had a normal performance status (KPS 100), while 15% of the patients had effort in normal activity (KPS 80 or lower). The most common tumor site was oropharynx followed by the oral cavity. All patients had an advanced tumor stage (III or IV) and 75% had regional metastasis at diagnosis.

**Table 1:** Patient, tumor, and nutritional data at diagnosis for all patients and divided into patients who survived 5 years or deceased in 5 years

Groups	All patients	5-year survivors	5-year non-survivors	*p* value
No. of subjects	128	80	48	
Age (years)				
Mean (SD)	61.4 (11.2)	58.2 (10.7)	66.7 (10.0)	<.001
Median (range)	60 (35–87)	57 (35–81)	66 (51–87)	
Gender, no. (%)				
Male	87 (68)	54 (68)	33 (69)	
Female	41 (32)	26 (33)	15 (31)	1.0
Performance status^a^				
100	62 (48)	47 (59)	15 (31)	
90	47 (37)	27 (34)	20 (42)	
80	15 (12)	4 (5)	11 (23)	
70	4 (3)	2 (3)	2 (4)	< .001
Tumor site				
Oropharynx	74 (58)	49 (61)	25 (52)	
Oral cavity	41 (32)	18 (23)	23 (48)	
Unknown primary	10 (8)	10 (13)	0	
Hypopharynx	2 (2)	2 (3)	0	
Nasopharynx	1 (1)	1 (1)	0	.006
T classification				
T0	10 (8)	10 (13)	0	
T1	17 (13)	14 (18)	3 (6)	
T2	32 (25)	22 (28)	10 (21)	
T3	20 (16)	14 (18)	6 (13)	
T4	49 (38)	20 (25)	29 (60)	< .001
N classification				
N0	32 (25)	20 (25)	12 (25)	
N1	34 (27)	19 (24)	15 (31)	
N2	49 (38)	35 (44)	14 (29)	
N3	13 (10)	6 (8)	7 (15)	1.0
Stage				
III	33 (26)	24 (30)	9 (19)	
IV	95 (74)	56 (70)	39 (81)	.23
Weight loss^b^ (%), mean (SD)	3.15 (4.77)	2.22 (4.04)	4.71 (5.49)	.002
Weight (kg)	75.1 (13.8)	77.1 (13.9)	71.9 (13.1)	.046
Height (cm)	173.5 (8.4)	174.0 (8.0)	172.7 (9.0)	.59
Body mass index (kg/m^2^)	24.9 (3.8)	25.3 (3.5)	24.1 (4.1)	.13
Resistance (Ω)	512 (79)	505 (73)	523 (87)	.27
Reactance (Ω)	52.3 (11.0)	53.9 (9.6)	49.5 (12.6)	.006
Fat-free mass percent (%)	77.6 (7.4)	77.1 (6.3)	78.4 (8.9)	.22
Fat-free mass index (kg/m^2^)	19.2 (2.6)	19.5 (2.6)	18.7 (2.7)	.11
Phase angle (°)				
Mean (SD)	5.85 (0.98)	6.13 (0.98)	5.38 (0.79)	< .001
Median (range)	5.91 (4.06–8.45)	6.22 (4.06–8.45)	5.32 (4.08–7.95)	
Standardized phase angle (SD)				
Mean (SD)	− 0.013 (1.043)	0.200 (1.059)	− 0.367 (0.921)	.002
Median (range)	0.035 (− 2.35–2.58)	0.28 (− 2.17–2.51)	− 0.29 (− 2.35–2.58)	

^a^Karnofsky performance status

^b^Unintentional weight loss 6 months before diagnosis

The patients’ mean weight at diagnosis was 75.1 kg, the mean BMI 24.9 and the mean unintentional weight loss 6 months before diagnosis was 3.15%.

The overall 5-year survival for all patients was 62.5%. When comparing patients surviving 5 years with non-survivors, there were significant differences in: age, performance status, tumor site, T classification, weight loss before diagnosis, weight, reactance, PA, and SPA (Table 1).

### Univariable survival analysis

Univariable Cox regression analyses on the overall survival was performed for patient characteristics, tumor, and nutritional factors (Table 2). Age was a significant factor for survival (hazard ratio 1.075 per year, *p* < .001) in contrast to gender. Performance status (KPS) was a significant prognostic factor for survival (hazard ratio 1.85 per step in the ordinal scale, *p* < .001). Tumor site was not a significant factor for survival when dividing it in five tumor sites (hazard ratio 1.35 per step, *p* = .31), but significant when divided it in oral cancer and non-oral cancer (hazard ratio 3.90, *p* < .001, Table 2). Tumor T classification was a significant prognostic factor for survival (hazard ratio 1.88, *p* < .001). Unintentional weight loss (hazard ratio 1.066, *p* = .001), weight (hazard ratio 0.975, *p* = .005), height (hazard ratio 0.78, *p* = .005), and BMI (hazard ratio 0.913, *p* = .010) were all significant factors for survival.

**Table 2:** Uni- and multivariable Cox regression analyses on the overall survival for patient characteristics, tumor, and nutritional factors

	Univariable analyses			Multivariable analysis		
	*n*	HR (95% CI)	*p*	*n*	HR (95% CI)	*p*
Age (years)	128	1.075 (1.051–1.100)	< .001	128	1.030 (1.000–1.062)	.050
Gender						
Male	87	1				
Female	41	0.95 (0.57–1.59)	.85			
Performance status^a^	128	1.85 (1.43–2.43)^c^	< .001	128	1.47 (1.14–1.90)^c^	.003
100	62	1				
90	47	1.64 (0.94–2.87)	.085			
80	15	5.40 (2.77–10.54)	< .001			
70	4	4.08 (1.40–11.88)	.010			
Tumor site				128	NS	NS
Non-oral	87	1				
Oral	41	3.90 (2.04–7.47)	< .001			
T classification	128	1.88 (1.48–2.39)^c^	< .001	128	1.49 (1.15–1.94)^c^	.003
T0	10	no deaths				
T1	17	1				
T2	32	1.29 (0.45–3.72)	.64			
T3	20	2.16 (0.75–6.21)	.15			
T4	49	4.42 (1.74–11.24)	.002			
N classification	128	0.85 (0.66-1.09)^c^	.20			
N0	32	1				
N1	34	0.88 (0.47–1.63)	.68			
N2	49	0.51 (0.27–.95)	.033			
N3	13	1.08 (0.48–2.43)	.86			
Stage						
III	33	1				
IV	95	1.35 (0.76–2.39)	.39			
Weight loss^b^ (%)	128	1.066 (1.025–1.107)	.001	128	NS	NS
Weight (kg)	128	0.975 (0.958–.992)	.005			
Height (cm)	128	0.78 (0.65-.93)	.005			
Body mass index (kg/m^2^)	128	0.913 (0.852–.978)	.010	128	NS	NS
Resistance (Ω)	128	1.002 (0.999–1.005)	.29			
Reactance (Ω)	128	0.955 (0.931–.979)	< .001			
Fat-free mass percent (%)	128	1.034 (0.996–1.073)	.078			
Fat-free mass index (kg/m^2^)	128	0.912 (0.831–1.001)	.052			
Phase angle (°)	128	0.47 (0.36–.62)	< .001	128	0.69 (0.50–.96)	.026
Standardized phase angle (SD)	128	0.66 (0.52–.84)	< .001			

*HR* hazard ratio, *CI* confidence interval, *NS* not significant with significance level 0.05 in the multivariable analysis

^a^Karnofsky Performance Status

^b^Unintentional weight loss 6 months before diagnosis

^c^The overall hazard ratio for the ordered categorical variables corresponds to the hazard ratio for each step in the ordinal scale

### BIA

Impedance measures resulted in a mean resistance value of 512 Ω (Table 1) and it was not a significant prognostic factor for survival (Table 2). The mean reactance value was 52.3 Ω (Table 1), and reactance was a significant prognostic factor for survival (hazard ratio 0.955, *p* < .001, Table 2). The mean fat-free mass percent was 77.6% and it was not a significant factor for survival (hazard ratio 1.034, *p* < .078). FFMI was 19.2 kg/m^2^ on average and it was not a significant factor for survival (hazard ratio 0.912, *p* = .052).

**Fig. 1:**
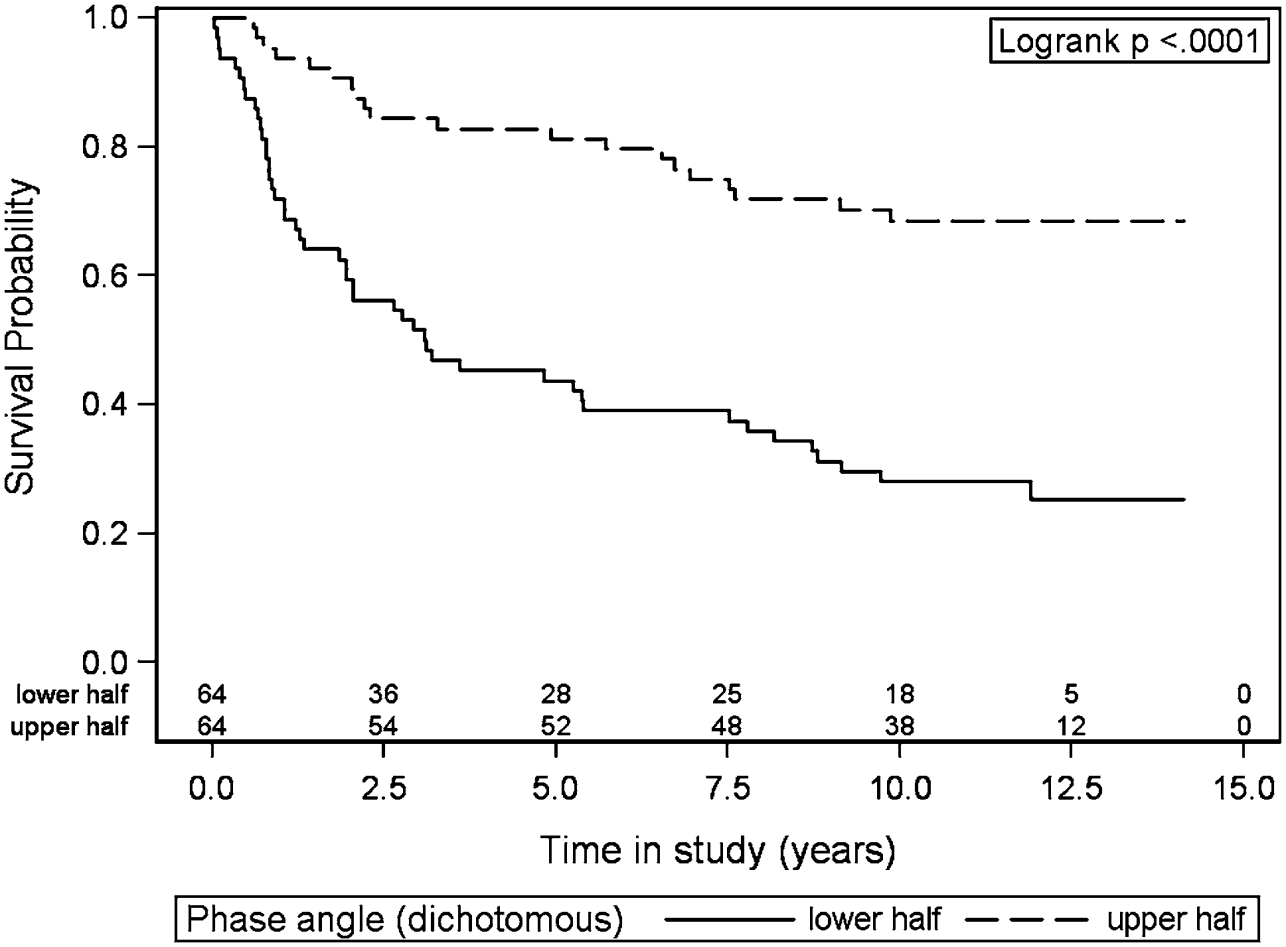
Overall survival probability for patients with phase angle values in the lower half versus in the upper half (the median phase angle value was 5.91°)

**Fig. 2:**
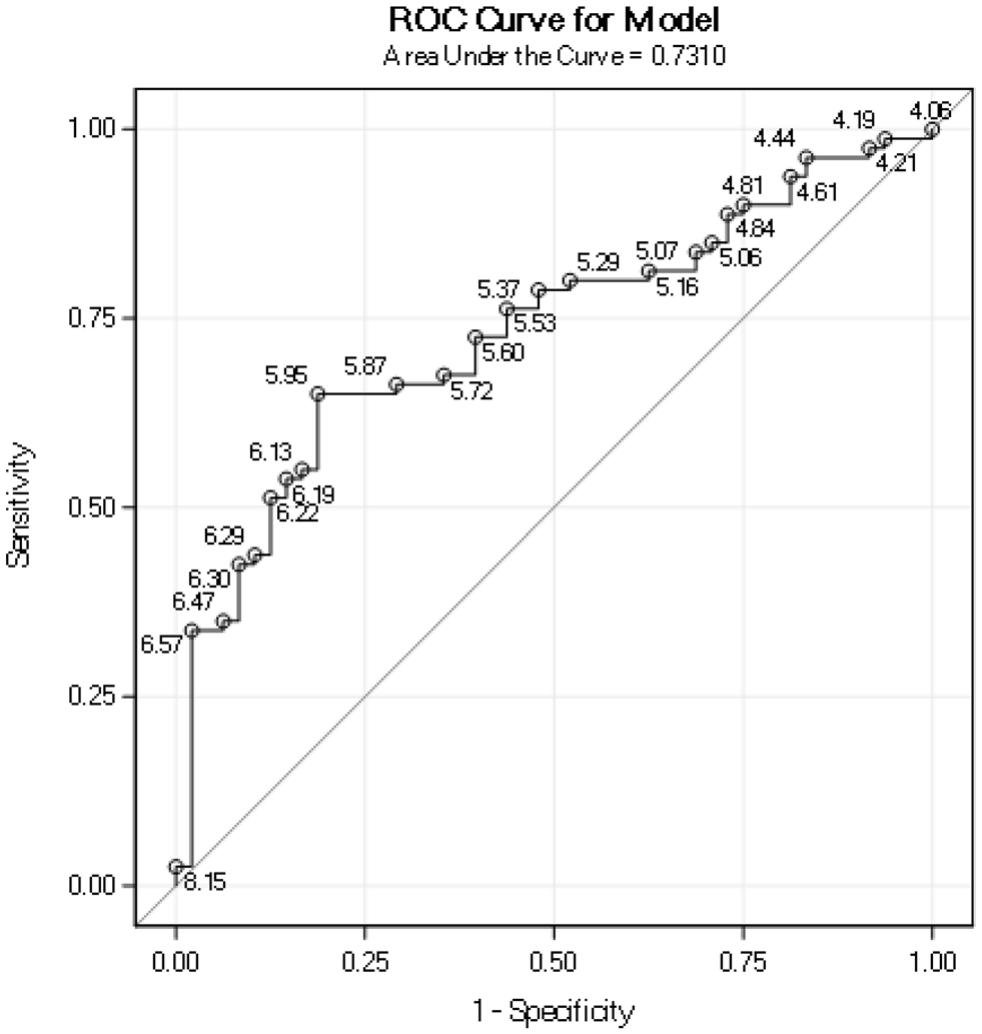
Receiver operating characteristic (ROC) curve of how the phase angle predicts 5-year survival. Points labeled by the phase angle value

The mean PA for all patients was 5.85° and the median PA was 5.91° (Table 1). PA was a significant prognostic factor for survival (hazard ratio 0.47, *p* < .001, Table 2). Patients with the lower half of PA values had significantly shorter survival than did the patients with higher values (median PA 5.91°, *p* < .001, Fig. 1). An ROC curve of how PA predicts 5-year survival gave an AUC of 0.73 (Fig. 2). The cut-point PA value that gave the most accurate prediction of 5-year survival was 5.95° (Fig. 2, with 70.3% correct predictions, sensitivity 64%, and specificity 81%). Among patients with PA ≤ 5.95, 43% survived after 5 years compared to 85% for patients with PA > 5.95°, as shown in Table 3. The most common cause of death in both groups was the initial cancer (Table 3).

**Table 3:** Comparison of patients with PA ≤ 5.95 and PA > 5.95 with regard to survival and different causes of death

Group	PA ≤ 5.95	PA > 5.95
Subjects, *n*	68	60
Survived 5 years *n* (%)	29 (43)	51 (85)
Deceased in 5 years	39 (57)	9 (15)
Of the treatment	8 (12)	0
Of the initial cancer	20 (29)	6 (10)
Of other disease	9 (13)	3 (5)
Of unknown cause	2 (3)	0

*PA* Phase angle

The mean SPA was − 0.013 SD (Table 1). SPA was a significant prognostic factor for survival (hazard ratio 0.66, *p* < .001, Table 2). Patients with the lower half of SPA values had significantly shorter survival than patients with higher values (*p* < .001). The AUC for SPA to predict 5-year survival was 0.66.

### Multivariable analysis

Multivariable analysis was performed including the significant factors from the univariable analysis: age, performance status, tumor site (oral vs non-oral), T classification, weight loss before diagnosis, BMI, and PA (Table 2). Weight and height were also significant factors but were not possible to include in the multivariable analysis together with BMI because both are used in the calculation of BMI. BMI is well known to correlate to PA and was therefore included [13, 14]. In analogy reactance was not included in the multivariable analysis because it is used in the calculation of PA. Both SPA and PA were significant but because both are calculated from the resistance and the reactance, only one of them could be included. Because PA was the main variable of the study and because SPA (unlike PA) is calculated from age and BMI, both of which are included in the multivariable analysis, PA was included. Significant factors for the overall survival in the multivariable analysis were: age (hazard ratio 1.030, *p* = .050), performance status (hazard ratio 1.47, *p* = .003), T classification (hazard ratio 1.49, *p* = .003), and PA (hazard ratio 0.69, *p* = .026, Table 2).

## Discussion

PA and SPA have been identified as prognostic factors for survival in different cancers and other severe diseases; the current study aimed to investigate whether PA and SPA are prognostic factors for survival in advanced HN cancer. BIA has been introduced in clinical practice to measure body composition. It is easily performed and can be done repeatedly at regular out-clinic visit during and after treatment. The test only requires approximately 3–5 min. FFM and FFMI may be assessed, which have been identified as important factors for malnutrition and survival [9, 23]. PA can easily be calculated at the same time.

The current study population included 128 patients with advanced HN cancer; the majority had oropharyngeal and oral cancer. Laryngeal cancer patients were not possible to include due to another ongoing study. The mean age was 61 years and the patients had a normal weight at diagnosis, with an average BMI of 24.9. The overall 5-year survival was comparably high for patients with advanced HN cancer, at 63% [24]. Age and performance status were significant prognostic factors for survival, as identified in previous studies [25, 26]. Tumor site (when divided in oral and non-oral cancer) and tumor T classification were significant prognostic factors for survival, as is well established [2].

Examining nutritional aspects, unintentional weight loss before diagnosis, weight, height, and BMI at diagnosis were significant factors for survival at univariable analyses; however, not at multivariable analysis. Previous studies have found significantly worse prognosis for malnourished patients, as assessed by lower BMI and weight loss [6, 27, 28]. Regarding results from BIA, and first FFMI, higher FFMI was associated with higher survival, but not significantly. A previous study of mixed cancer patients found, in contrast to the current study, that patients with a high FFMI had a significantly better prognosis than did patients with a low FFMI [8].

The main aim of the current study was to study PA as a prognostic factor for survival. The median PA at diagnosis for all patients was 5.91°, which was similar as in a previous study of mixed cancer patients with a median PA at 5.95° [29]; however, in two studies, one on breast cancer [15] and one on colorectal cancer [30], the median PA was lower at 5.6°; and one study on patients with HN cancer identified a lower mean PA of 5.04° [31]. In the current study, PA was a significant prognostic factor for survival at uni- and multivariable analyses. This is considered a main finding of this study, because it shows that PA is a potentially useful prognostic factor in HN cancer. Previous studies of patients with different cancers, including one of HN cancer, also support this finding, and found that PA was a significant factor for survival [30–32].

To investigate the prognostic value of PA, an ROC curve was created (Fig. 2). The AUC was 0.73, which is fair accuracy for a test. The PA value that resulted in the most accurate prediction of 5-year survival was 5.95°. When this value was used as a cutoff, 47% of the patients were above the value and 53% were at or below. Among patients with PA ≤ 5.95° (“worse prognosis group”), the 5-year survival was 43% compared to 85% for patients with PA > 5.95° (“better prognosis group”). Examining cause of death for the worse prognosis group, most patients died of the initial cancer; however, many patients had treatment-related deaths (refer to Table 3). In the better prognosis group, most patients died of the initial cancer and none died from their treatment. One can conclude that patients in the worse prognosis group die both from the cancer and the treatment, and whereas these patients probably benefit from having intensive cancer treatments, they require a careful monitoring and nutritional care during and after treatment. For the patients in the better prognosis group, their cancers were successfully managed according to existing treatment guidelines, leading to a relatively low risk of dying from the cancer and an even lower risk dying from the treatment. In summary, PA is a promising method to group patients with advanced HN cancer in different risk groups; however, further studies are needed before it can be used for treatment planning.

PA is known to be dependent on age, sex, ethnicity, and BMI [14], so to determine if the precision of PA in predicting survival was improved when correcting for these factors, SPA was used [13]. The mean SPA was − 0.013 SD for all patients, which was higher than expected for a group of patients with advanced HN cancer. (The mean SPA was only 0.013 SD = 0.5% lower than the healthy German reference population in the study by Bosy-Westphal et al. [13]). SPA was a significant prognostic factor for survival. A previous study of cancer patients correcting PA for age and sex, also identified SPA to be a significant prognostic factor for survival [17]. In the current study, three different factors derived from the BIA variables’ resistance and reactance were highlighted: FFMI, PA, and SPA. Comparing these factors regarding how well they predicted survival in HN cancer, PA and SPA were both significant at the 0.05 level in contrast to FFMI (Table 2). Comparing PA and SPA, the AUC of the ROC curve for 5-year survival was 0.66 for SPA and 0.73 for PA, i.e., SPA was less good in predicting 5-year survival than PA. One explanation for that PA was better than SPA in predicting survival may be found when analyzing the reference values. Increased age and lower BMI were two significant negative factors for survival in the current study and correcting for these factors with SPA compared to PA thus impairs the prediction of survival. To our knowledge, no study has been performed examining SPA as a predictor for survival in advanced HN cancer patients, and further studies of SPA in HN cancer are needed.

The number of patients in the study was 128, making it, to our knowledge, the largest study of the prognostic value of PA and SPA in HN cancer. There are some factors not included in the study that would be interesting to explore in relation to PA and SPA, including smoking and HPV status, CRP and albumin. One limitation of BIA is that it occasionally gives erroneous values despite retesting a patient, which in this study accounted for 4% of the values (six patients). Both resistance and reactance values were affected, and we observed both extremely high and low SPA (four patients had SPA > 3 SD and two < − 3 SD). The 5-year survival for these patients were 25% (1/4) for patients with extremely high SPA and 50% (1/2) for patients with extremely low SPA. Different factors may contribute to BIA measurement problems in clinical practice, including patients at the extremes of BMI ranges (outside 16–34 kg/m^2^) or with abnormal hydration [33]. We approached this problem by excluding these patients from analysis.

## Conclusions

In this study of patients with advanced HN cancer, low values of bioelectrical PA and SPA at diagnosis were significantly associated with shorter overall survival in univariable analyses, along with higher age, oral cancer tumor site, higher T class, worse performance status, and lower weight. Age, performance status, T classification, and PA were significant prognostic factors for survival in multivariable analysis. A PA cutoff value at 5.95° provided the best prediction of 5-year survival. PA and SPA are promising prognostic tools in HN cancer and further studies are needed to examine how useful these factors are in treatment planning.
